# Prediction of Cholecystokinin-Secretory Peptides Using Bidirectional Long Short-term Memory Model Based on Transfer Learning and Hierarchical Attention Network Mechanism

**DOI:** 10.3390/biom13091372

**Published:** 2023-09-11

**Authors:** Jing Liu, Pu Chen, Hongdong Song, Pengxiao Zhang, Man Wang, Zhenliang Sun, Xiao Guan

**Affiliations:** 1 College of Information Engineering, Shanghai Maritime University, Shanghai 201306, China; jingliu@shmtu.edu.cn (J.L.); chenpu@stu.shmtu.edu.cn (P.C.); 2 School of Health Science and Engineering, University of Shanghai for Science and Technology, Shanghai 200093, China; songhd@usst.edu.cn; 3 Joint Center for Translational Medicine, Southern Medical University Affiliated Fengxian Hospital, Shanghai 201499, China; lyn_zhangxl@sumhs.edu.cn (P.Z.); lyn_wangm@sumhs.edu.cn (M.W.)

**Keywords:** cholecystokinin, CCK-secretory peptides, transfer learning, SMILES enumeration, hierarchical attention network, BiLSTM

## Abstract

Cholecystokinin (CCK) can make the human body feel full and has neurotrophic and anti-inflammatory effects. It is beneficial in treating obesity, Parkinson’s disease, pancreatic cancer, and cholangiocarcinoma. Traditional biological experiments are costly and time-consuming when it comes to finding and identifying novel CCK-secretory peptides, and there is an urgent need to develop a new computational method to predict new CCK-secretory peptides. This study combines the transfer learning method with the SMILES enumeration data augmentation strategy to solve the data scarcity problem. It establishes a fusion model of the hierarchical attention network (HAN) and bidirectional long short-term memory (BiLSTM), which fully extracts peptide chain features to predict CCK-secretory peptides efficiently. The average accuracy of the proposed method in this study is 95.99%, with an AUC of 98.07%. The experimental results show that the proposed method is significantly superior to other comparative methods in accuracy and robustness. Therefore, this method is expected to be applied to the preliminary screening of CCK-secretory peptides.

## 1. Introduction

Cholecystokinin (CCK) is a gastrointestinal hormone that causes gallbladder contraction and was discovered and named by Ivy and Oldbery in 1928. CCK is a peptide hormone comprising 33 amino acids, released by the small intestinal mucosa I cell. CCK plays various roles in the organism, such as producing satiety to inhibit food intake, slowing gastric emptying, and stimulating pancreatic and gallbladder secretion production [1,2,3,4]. Cholecystokinin-8 and nerve growth factor (NGF) work together to maintain and repair the nervous system [5]. Su et al. [6] found that CCK has neurotrophic and anti-inflammatory effects and improves adverse effects, such as the inflammatory response and neuronal damage, in Parkinson’s disease patients. Numerous experiments have confirmed that CCK is a vital tumor growth factor in the digestive tract and has an apparent promoting effect on pancreatic and bile duct cancer [7,8]. In recent years, therapeutic peptides have emerged as an advanced and novel cancer treatment strategy that can treat many diseases [9,10,11]. Compared with traditional recombinant antibody therapies, peptide-based therapies are highly targeted, productive, less toxic, and easy to synthesize and modify [12]. CCK as a therapeutic peptide will also offer broad research prospects. CCK-secretory peptides can target the intestinal endocrine cell membrane CaSR to activate the Gq signalling pathway, promoting the secretion of CCK by the intestinal endocrine cells. High-quality CCK-secretory peptides have the advantages of safety, no toxic side effects, gastrointestinal digestive enzyme hydrolysis tolerance, and easy absorption. CCK-secretory peptides are suitable for developing food and drugs as functional ingredients or food base materials. The prediction of CCK-secretory peptides has a significant application value in developing foods, health foods, and drugs with functions such as delaying gastric emptying, promoting satiety, and weight loss.

In recent decades, biological researchers have conducted numerous experiments to study the substances that stimulate CCK secretion. It has been demonstrated that the primary nutrients stimulating CCK release are ingested fats and proteins, especially amino acids, peptides, and protein hydrolysates [13]. Daly Kristian et al. [14] demonstrated that Phe, Leu, and Glu induce CCK secretion via small-intestinal tissue experiments in mice. In pig jejunal tissue experiments, Leu, Ile, or a mixture of amino acids significantly increased CCK secretion [15]. Santos-Hernández et al. [16] found that VLLPDEVSGL and a derivative fragment, VLLPD, induced CCK release in gastrointestinal egg-white digestion. The above experiments require a high amount of time from biological researchers, with the help of numerous devices, to speculate whether peptides or amino acids have a facilitative effect on CCK. These tasks are tedious, labor-intensive, and costly. 

With the increasing therapeutic importance of CCK, there is an urgent need to develop efficient, accurate, and cost-effective predictive techniques for CCK-secretory peptides. The issues above have prompted us to propose a method to address the limitations of biological clinical experiments. Nowadays, numerous in silico techniques have been applied in various fields. Mol2vec [17] has been utilized in the field of chemistry to identify the vector representations of molecular substructures. Similarly, Jo et al. [18] employed message-passing neural networks for predicting SMILES chemical properties. In natural language processing (NLP), the word2vec [19] method has also been applied to the semantic search of relevant documents. Sunjaya et al. [20] used ARIMA and long short-term memory (LSTM) methods to predict the number of positive COVID-19 cases in Indonesia, and the results showed that the LSTM model outperformed the ARIMA model. Han et al. [21] proposed an LSTM model that combines input and hidden layer attention mechanisms for long-term streamflow prediction in the environmental domain. Liu et al. [22] introduced the MolRoPE–BERT framework, which integrates an effective position-encoding method for capturing sequence position information and a pre-trained BERT model for molecular property prediction. However, these in silico techniques still face challenges, as they require a large number of training data and may struggle to achieve a good prediction performance on small-scale datasets.

This paper describes a method to predict CCK-secretory peptides with high accuracy. The sequences of peptides associated with CCK secretion are efficiently characterized as SMILESs, and data augmentation is achieved via SMILES enumeration. Based on this, a BiLSTM model based on transfer learning and hierarchical attention networks was developed to predict CCK-secretory peptides. In the training phase, a two-stage transfer learning strategy was adopted to train the model, to prevent overfitting. The data augmentation and transfer learning strategy solves the problem of the scarcity of CCK-secretory peptide data, while improving the performance and generality of the model. The model has a more significant performance and accuracy compared with machine learning models based on the AAC and DPC representations and similar transfer learning models. Therefore, the proposed method is expected to be applied in the initial screening of CCK-secretory peptides. Molecular docking [23,24] helps us to understand the interactions between molecules, and the combination of the proposed method and molecular docking is conducive to developing CCK-secretory peptide foods and drugs.

## 2. Materials and Methods

### 2.1. Construction of Datasets

In this work, two datasets were used to construct the model, named as the source dataset and target dataset. The details of the datasets are shown in Table 1.

Positive samples are denoted by $C^{+}$, and negative samples are denoted by $C^{-}$. Therefore, the whole dataset can be denoted as C.

(1) $$C=C^{+}\cup C^{-},$$

In addition, there is no overlap between the positive and negative dataset.

(2) $$\emptyset =C^{+}\cap C^{-},$$

Source dataset: To train a source task prediction model, four high-quality absorption, distribution, metabolism, excretion, and toxicity (ADMET) datasets were collected from ADMETlab [25,26]. The source datasets contain pgb-sub, DILI, and SR-MMP, which have some similarities to the target dataset, in that they are all drug-related datasets. The CCK-secretory peptides can also be used in targeted therapy peptide drug development. The datasets with partial similarity are conducive to the transfer of transfer learning model knowledge. And these datasets have different sizes, ranging from 467 to 1840, which can better explore the differences between datasets.

Target dataset: This dataset contains 99 data obtained from 37 papers investigating CCK-secretory peptide activity. Among them, the number of samples with effective peptides was 54, and the number of samples with ineffective peptides was 45. The sample distribution in the dataset is relatively balanced, which can prevent the overfitting of the model caused by an unbalanced dataset, to some extent.

Each dataset is randomly divided into training and testing datasets, using a hierarchical strategy, with ratios of 0.8 and 0.2, respectively. Therefore, the sample distribution in the training and test datasets is the same for an original dataset. All experiments were repeated five times, using different random seeds, to minimize the impact of the sample distribution on the model performance. The model was trained on the training dataset, and the parameter selection was adjusted to achieve a better model performance. The average of the five repeated experiments on the test dataset was calculated as the final model performance. The proposed model and comparison method were evaluated according to sensitivity (Sen), specificity (Spe), accuracy (Acc), Matthews correlation coefficient (MCC), and the area under the curve (AUC).

### 2.2. Feature Representation of Peptides

Efficient feature representation is essential for constructing predictive models and, therefore, requires the generation of corresponding features for each peptide sequence. The feature representation method has an important impact on the performance of the prediction model. This study used three different features to develop models based on machine learning and deep learning techniques. The machine learning model uses the currently popular machine learning peptide representation, including amino acid composition and dipeptide composition. The deep learning model uses a SMILES to characterize the sequence of the peptide. Below is a brief description of the different feature representation methods and principles.

#### 2.2.1. Amino Acid Composition

Characterizing amino acids or protein sequences as AAC is one of the simplest methods available for feature extraction. The AAC descriptor represents the frequency of standard amino acids occurring in protein sequences [27]. As all peptides are composed of 20 standard amino acids, the AAC can be expressed as a vector of dimension 20, and we calculate the AAC as follows.

(3) $$AAC(i)=\frac{aa(i)}{L},$$

where AAC(i) is the abundance of the ith amino acid, aa(i) is the number of occurrences of the ith amino acid, and L is the length of the peptide.

#### 2.2.2. Dipeptide Composition

Dipeptide composition (DPC) can express more information than AAC and represent the local order of amino acid sequence pairs [28]. We calculated all possible dipeptides (i.e., amino acid sequence pairs, e.g., AA, AC, etc.) with a dimension 400 (20 × 20) vector, to represent the DPC. The DPC is calculated as follows.

(4) $$DPC(i)=\frac{dp(i)}{L-1},$$

where DPC(i) is the frequency of occurrence of the ith dipeptide, dp(i) is the number of circumstances of the ith dipeptide, and L is the length of the peptide.

#### 2.2.3. Simplified Molecular Input Line Entry System

The Simplified Molecular Input Line Entry System (SMILES) is a chemical notation system for modern chemical information processing [29]. The SMILES is based on the principles of molecular graph theory and allows for a strict structural specification, by using a minimal and natural syntax. The SMILES naming rules are simple and easy to understand for researchers, and the system is considered to be the best compromise between human and machine chemical notation to date. Computationally, the SMILES interprets chemical molecules speedily and compactly, thus meeting machine time and space-saving goals. The SMILES is based on a computer-language-parsing approach, and our conversion of peptide sequences to the SMILES will significantly improve the data processing efficiency.

### 2.3. Data Augmentation

Deep learning has developed rapidly in recent years, and the computational power has increased greatly. However, deep learning usually requires a large number of samples for training, to achieve better generalization. Data augmentation is a good idea to solve this problem. Data augmentation refers to the method of increasing the number of data by adding small changes to the existing data, or creating new synthetic data from the existing data. This strategy solves the data imbalance problem, while expanding the dataset, improves the performance and generalization of the model, and prevents the model from overfitting, to some extent [30].

**Figure 1:**
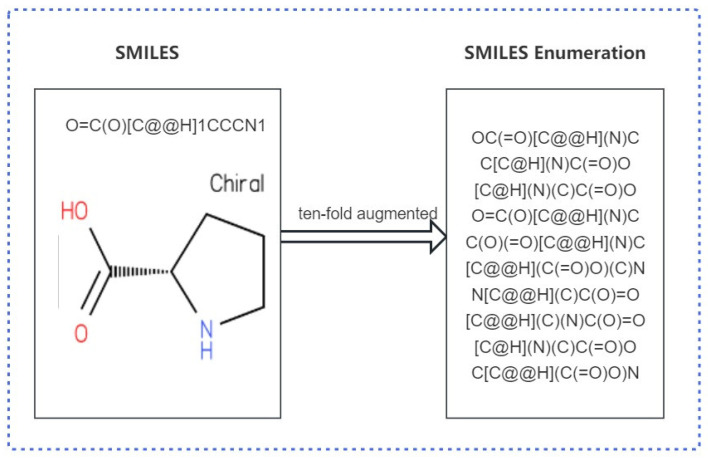
SMILES enumeration enables data augmentation. One SMILES is ten-fold augmented to obtain ten different SMILESs.

This study used SMILES enumeration for data augmentation. SMILES enumeration uses a Python script to generate multiple SMILESs, by changing the order of atoms using the cheminformatics library RDKit, wherein different atom orders lead to different SMILESs, thus achieving data augmentation [31]. An example of the use of a SMILES for data augmentation is shown in Figure 1.

### 2.4. Machine Learning Models

To demonstrate the predictive power of a hierarchy-attention-network-based BiLSTM deep learning model for CCK-secretory peptides, we built some classical machine learning models for comparison. Specifically, in this study, we characterized the peptide sequences as AAC and DPC forms, respectively, and then built machine learning classifiers based on the AAC and DPC representations using SVM light and scikit-learn from the Python library, respectively, including support vector machines (SVM), random forest (RF), multilayer perceptron (MLP), k-nearest neighbor (KNN), gradient-boosting decision tree (GBDT), and extreme gradient boosting (XGBoost). The relevant parameters of different classifiers were adjusted in the modeling process, and the best parameters are reported.

### 2.5. Transfer Learning Models

The transfer learning model used in this work is a bidirectional long and short-term memory network model based on a hierarchical attention network. SMILESs encoded after data enhancement are fed into this model. A BiLSTM is used to aggregate the information and extract the best features, through a new hierarchical attention network. Following are brief descriptions of each part of the model. 

#### 2.5.1. Bidirectional Long and Short-Term Memory

**Figure 2:**
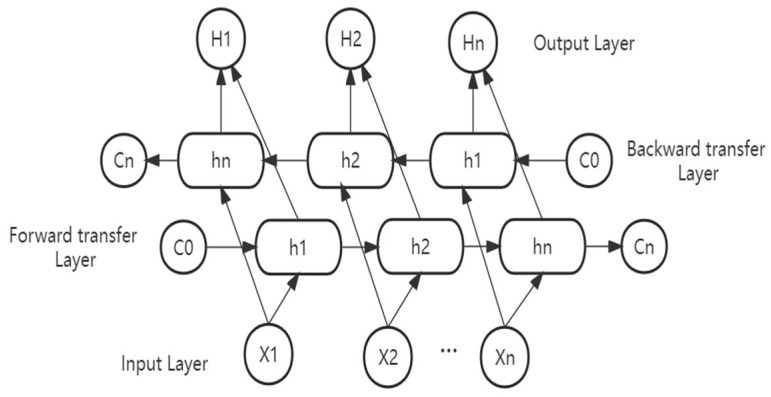
BiLSTM structure diagram. X_n_ represents the input, H_n_ represents the output, h_n_ is the hidden layer vector, and C_n_ is the cell state at the moment n.

The bidirectional long and short-term memory network comprises forward long and short-term networks and backwards long and short-term networks. BiLSTM and LSTM are variants of RNN. Researchers solved the problems of RNN gradient explosion and the poor ability to rely on information over long distances by adding gating mechanisms (forget gate, input gate, output gate) to preserve information long term, thus proposing LSTM [32]. The sequences of peptides are input to two LSTMs of BiLSTM in forward and reverse order, respectively, for feature extraction, and the word vector formed via the stitching of the two output vectors (i.e., the extracted feature vectors) is used as the final feature expression of the word. The model design concept of BiLSTM is to make the feature data obtained at the time t have the information between the past and future simultaneously. It is worth mentioning that the two LSTM neural network parameters in BiLSTM are independent, and they only share the word-embedding word vector list. The structure of BiLSTM is shown schematically in Figure 2.

The LSTM model can better capture the longer-distance dependencies. This is because LSTM uses a gating mechanism to determine which information should be remembered and which should be forgotten. However, modelling sentences with LSTM also has a problem: it cannot encode information from back to front. In this study, we used BiLSTM to aggregate the sequence information of peptides. BiLSTM allows for the better capture of bidirectional textual semantic dependencies.

#### 2.5.2. Hierarchical Attention Network Mechanism

The attentional mechanism mimics the internal processes of biological observation behavior and is a mechanism that combines internal experience with external sensation, to improve the precision of observation in certain regions [33]. This mechanism provides a higher scalability and robustness, by focusing on important information based on the size of the weights, and continuously adjusting the weights so that important information can be selected, even under different circumstances. In this study, the hierarchical attention network mechanism is used to focus on more critical features gradually.

The hierarchical attention network (HAN) consists of several components: a word sequence encoder, word-level attention layer, sentence encoder, and sentence-level attention layer [34]. The embedding word layer first transforms the input text into vector form. The BiLSTM layer extracts the text features, the global information of the text is obtained using the self-attention layer and, finally, the fusion and pooling are performed by the fusion and pooling layers. Finally, the fusion and pooling layers are used to fuse and pool the text. After the splicing layer, the fully connected layer, and the SoftMax, the implication relationship in the text is obtained, to achieve the classification of the text. The attention layer is calculated as follows:

(5) $$u_{p_{i}}=\tanh(W_{W}h_{p_{i}}+b_{w}),$$

(6) $$\alpha_{p_{i}}=\frac{\exp(u_{P_{i}}^{T}u_{w})}{\sum_{i}\exp(u_{P_{i}}^{T}u_{w})},$$

(7) $$V_{P}=\sum_{i}\alpha_{p_{i}}h_{p_{i}},$$

where $W_{w}$ and $b_{w}$ are the adjustable weights and bias terms of the attention model, tanh is the activation function, $h_{p}{}_{{}_{i}}$ is the output of the BiLSTM layer, $u_{w}$ is the weight value, and the calculated result $\alpha_{p_{i}}$ indicates the important information of each word in the sentence; $V_{P}$ is the output vector of the text after the attention model calculation. Similarly, the output vector $V_{h}$ of the hypothetical text after the attention model calculation can also be calculated.

#### 2.5.3. Design of Transfer Learning Models

Transfer learning is an approach to improving a learner by transferring information from one domain to a related domain, which can reduce training costs and overcome the problems of data scarcity and time-consuming training [35]. Transfer learning can be simply defined as: given a source domain $D_{s}$, a learning task $T_{s}$, and corresponding target domains $D_{t}$ and $T_{t}$, the effect of improving the target prediction function $f_{t}(\cdot )$ in $D_{t}$ by the learning knowledge already available in $D_{s}$ and $T_{s}$.

**Figure 3:**
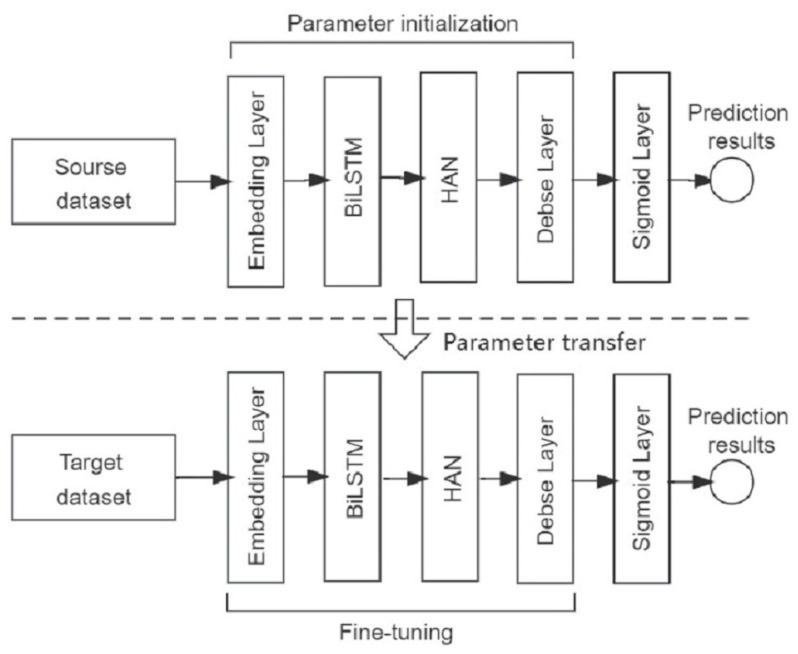
Process diagram of transfer learning.

The transfer learning method used in this study is parametric transfer, and the transfer learning model is built in two phases using this method. In the first phase, the source dataset is fed into the model for source task pre-training and prediction. In the second stage, the model parameters learned from the source domain are migrated to the target dataset, and the parameters are adjusted slightlym to make the model applicable to the target task. The transfer learning process in this study is shown in Figure 3.

### 2.6. Our Approach

**Figure 4:**
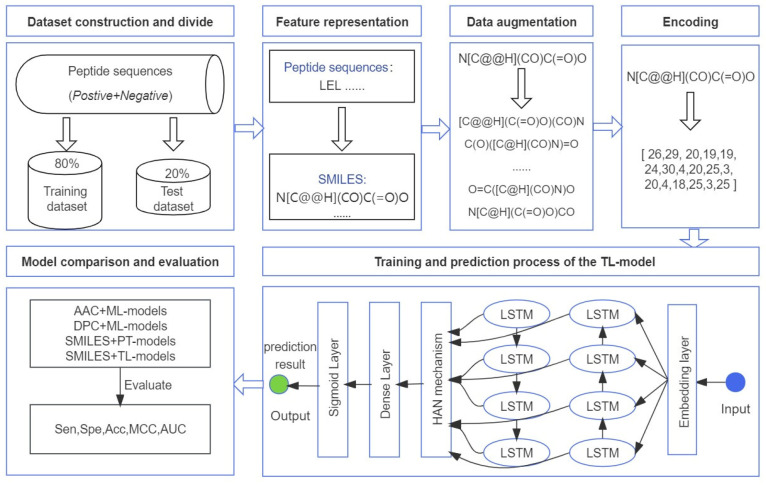
Flowchart of our proposed method. HAN: hierarchical attention network; ML: machine learning; PT: pre-training; TL: transfer learning.

The framework of our proposed method is shown in Figure 4. The amino acid and peptide sequences are randomly divided into training and test sets, in a 4:1 ratio. All peptide sequences are represented as SMILESs, and the target dataset is augmented via the implementation of a SMILES enumeration strategy. The SMILESs were then encoded, and the encoded features were used as the input for the transfer learning model mentioned previously. A BiLSTM prediction model, based on transfer learning and the hierarchical attention network mechanism, is used to accomplish the target task, which is named by us as TL-AHB.

Specifically, the target dataset was preprocessed using the SMILES enumeration technique, and the model was pre-trained on the source dataset using transfer learning. We solved the scarcity problem in the training data, while significantly improving the performance and accuracy of the model. Considering the large number of SMILESs of large length in the dataset, BiLSTM can better capture the textual semantic dependencies in both directions over longer distances. The hierarchical attention network, which progressively extracts features with higher weights, provides the most critical features for the model. Thus, the TL-AHB model combines the advantages of BiLSTM and HAN, which is important in improving the model’s performance. The use of transfer learning and data augmentation strategies allows us to obtain models with good predictive and generalization capabilities compared to traditional methods.

### 2.7. Performance Evaluation Metrics

This study investigates a prediction problem of CCK-secretory peptides, and this problem can be considered a binary classification problem. Therefore, five metrics widely used in binary classification tasks were used to evaluate the performance of the model, including the sensitivity (Sen), specificity (Spe), accuracy (Acc), Matthews correlation coefficient (MCC), and area under the curve (AUC). Each metric was calculated as follows:

(8) $$Sen=\frac{TP}{TP+FN},$$

(9) $$Spe=\frac{TN}{TN+FP},$$

(10) $$Acc=\frac{TP+TN}{TP+FP+TN+FN}\;,$$

(11) $$MCC=\frac{\left(TP\times TN)-(FP\times FN\right)}{\sqrt{\left(TP+FP)(TP+FN)(TN+FP)(TN+FN\right)}},$$

where TP represents the true positive number, TN represents the true negative number, FP represents the false positive number, and FN represents the false negative number. Sen, Spe, and Acc take values in the range (0, 1), and MCC takes values in the range (−1, 1). Sen and Spe measure the predictive ability of the classification predictor for positivity and negativity, respectively, and Acc and MCC are used to assess the overall performance of the predictor. ROC curves were plotted based on the false-positive and false-negative rates, and AUC was the area under the ROC curve.

## 3. Results and Discussion

To comprehensively evaluate the ability of our proposed method to predict CCK-secretory peptides, firstly, the amino acids of peptides were analyzed, and machine learning models based on AAC and DPC representations were built on the target dataset. Then, the performance of our proposed method was analyzed on the source and target tasks, respectively. Finally, some CCK-secretory peptide sequences with high similarity were used to verify the model’s predictive ability.

### 3.1. Compositional Analysis

**Figure 5:**
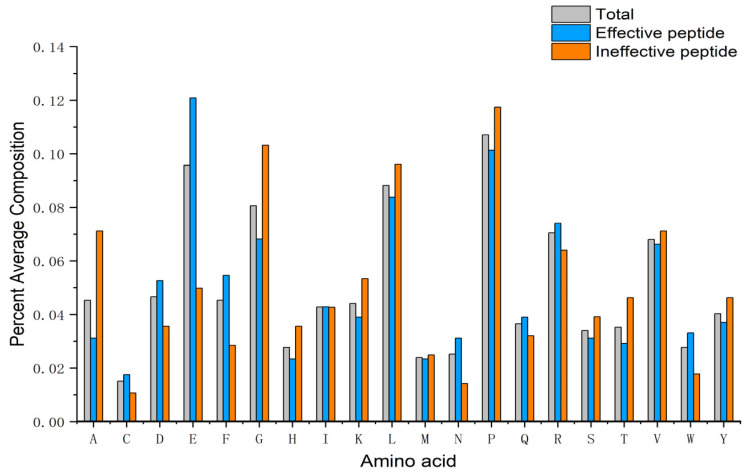
Bar plot showing the AAC percentage of CCK-secretory peptides.

Amino acid composition analysis is critical, because the two-terminal residues play a key role in the biological study of peptides. Firstly, the amino acid distributions in the active, the inactive, and all peptides need to be analyzed in the target dataset. The results of the analysis are shown in Figure 5, where the compositions of all 20 amino acids in the peptides associated with CCK secretion are compared and counted. Specific residues, including Glu, Phe, and Arg, were higher in the effective peptides than in the ineffective ones. In contrast, Ala, Gly, Leu, and Pro were higher in the ineffective peptides.

### 3.2. Analysis of Machine Learning Model Based on AAC and DPC Representations

Some peptide sequences are long, and their arrangement is complex, making it impractical to use the sequence directly for prediction; as commonly used characterization methods for peptide sequence prediction, AAC and DPC can simplify sequences, extract different features to replace complex sequences, and achieve interval prediction. Both have advantages in characterizing peptide sequences, and AAC features can represent a sequence with fewer feature dimensions and more straightforward calculations. On the other hand, dipeptide features contain the positional information of amino acids in protein sequences, making them more accurate in characterizing sequence information. We established machine learning models on the target dataset using six classic algorithms (SVM, RF, MLP, KNN, GBDT, XGBoost) to study the performance of different models, based on two representation methods. Each model has been adjusted and tested on the optimal parameters. These models were evaluated on the test set, and the experimental results are shown in Table 2 and Table 3.

Table 2 and Table 3 show the details of the performance comparison of the machine learning models based on AAC and DPC, respectively. It can be observed that the best performance is achieved using the GBDT algorithm, with 84.62% for Sen, 85.71% for Spe, 85.19% for Acc, 70.33% for MCC, and 85.16% for AUC in the machine learning prediction model with the AAC representation approach. In the machine learning prediction model with the DPC representation approach, the SVM algorithm model showed the best performance, but Acc was only 57.14%, and AUC was 59.09%. It is obvious that the performance of the machine learning prediction model based on the AAC representation approach is better than that of the prediction model based on the DPC representation approach, overall. Both representation methods have their limitations. The AAC feature disregards the positional information of the amino acids in the sequence, while the DPC feature has a higher dimensionality, making the calculations relatively complex. Due to data scarcity, both methods show a poor performance. Although the former has shown a better prediction ability, the model still has huge room for improvement when applied to predict CCK-secretory peptides. This also motivates the creation of our proposed method.

### 3.3. SMILES Augmentation Times on Model Impact Analysis

**Figure 6:**
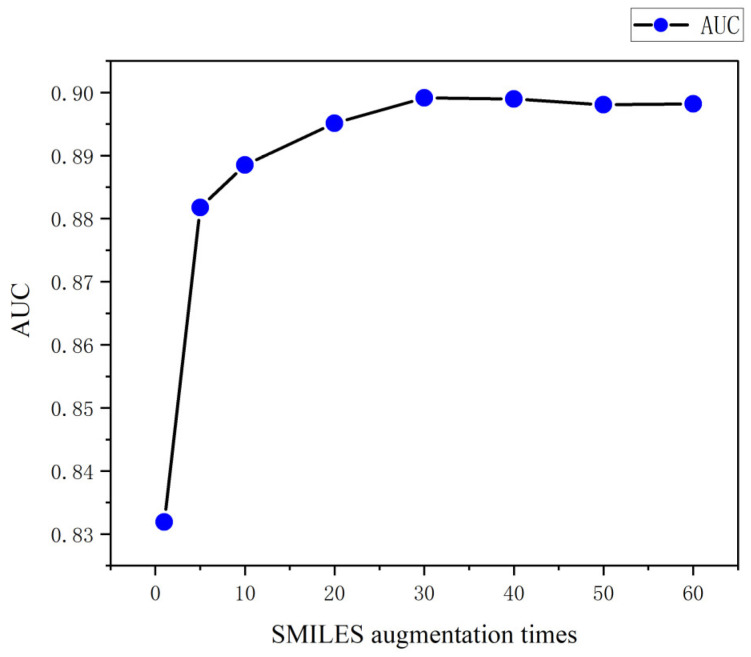
Trend of AUC with SMILES augmentation times.

The SMILES of one molecule may obtain tens or even hundreds of different SMILES expressions via data augmentation, and the amount of data has a great impact on the model performance, so we analyzed the relationship between the number of SMILES augmentations and the model performance. The impact of different augmentation times (1, 5, 10, 20, 30, 40, and 50) on the model performance was evaluated on the source dataset using pgb-sub as an example. As shown in Figure 6, the AUC of the model is only about 81% when no data augmentation is applied to the SMILES. As the augmentation times increase, the AUC also increases, and the trend of performance improvement eventually levels off. In order to consider the performance and training time of the model, an optimal augmentation threshold needs to be determined for the SMILES. According to the results shown in Figure 6, the model levelled off at an augmentation time of 30, so the augmentation time of the SMILES was set to 30.

### 3.4. Analysis of Prediction Results of Source Tasks on Different Models

Then, we built four deep learning pre-trained models on the source dataset and used them for the prediction analysis of three datasets, DILI, pgb-sub, and SR-MMP. These four deep learning pre-trained models include HL, AHL, HB, and AHB. This set of experiments demonstrates the predictive power of the BiLSTM model based on SMILES representation and hierarchical attention network, where A denotes data enhancement using SMILES enumeration, and H denotes the use of the hierarchical attention network mechanism. The evaluation results of the source dataset on different pre-trained models are shown in Table 4.

As shown in Table 4, experimental control groups, with and without data augmentation, were set up in the pre-training experiments, to show the effect of SMILES enumeration on the pre-trained models. In the three source datasets, AHL improved the AUC by 2.04%, 4.99%, and 2.26%, respectively, compared with the HL model; AHB improved the AUC by 4.00%, 4.48%, and 1.66%, respectively, compared with the HB model. The performances of the models with data augmentation were all improved to some extent. On the other hand, BiLSTM also played a key role in model performance improvement. AHB improved the AUC by 2.17%, 1.74%, and 0.27% for the three datasets compared to the AHL model, respectively. As mentioned earlier, LSTM captures information through a gating mechanism and can only retain unidirectional information about the sequence, and cannot encode information from the reverse to the forward direction. BiLSTM, because of the introduction of reverse information, can be trained in both the forward and reverse directions. Despite the higher computational complexity, BiLSTM captures bidirectional textual semantic dependencies, learns global and local features, and can better aggregate peptide chain information. BiLSTM can provide a better performance in datasets containing a large number of long sequences.

### 3.5. Analysis of Prediction Results of Target Tasks on Different Models

After completing pre-training on the source dataset, four transfer learning models, including TL-HL, TL-AHL, TL-HB, and TL-AHB, were obtained by combining the transfer learning strategy and parameter fine-tuning, wherein TL denotes transfer learning. Finally, the four transfer learning models were used in the identification of CCK-secretory peptides. The performance evaluation of the models is shown in Table 5.

It is obvious that the BiLSTM model (TL-AHB) based on transfer learning and the hierarchical attention network mechanism showed the best performance in predicting CCK-secretory peptides. The sensitivity (Sen) was 90.97%, the specificity (Spe) was 98.92%, the accuracy (Acc) was 95.99%, the Matthews correlation coefficient (MCC) was 91.27% and, in particular, the area under the ROC curve reached 98.07%. Among the machine learning models based on AAC and DPC representations, the best model is GBDT, with Acc and AUC of 85.19% and 85.16%. TL-AHB improves Acc and AUC by 10.8% and 12.91% compared to the former. Among the pre-training models, the best-performing model is the AHB model on the SR-MMP dataset, with Acc and AUC of 83.81% and 91.13%, and TL-AHB has an Acc and AUC improvement of 12.18% and 6.94% over this model. According to the experimental results, it can be seen that TL-AHB shows a strong predictive ability and excellent stability in predicting CCK-secretory peptides after transfer learning.

### 3.6. Analysis of the Prediction Performance of Similar Sequences on the Model

To investigate the model’s predictive ability for some similar CCK-secretory peptide sequences, some similar CCK-secretory peptide sequences of known classes were selected to validate the model. Each peptide sequence was entered into the model and predicted 50 times, and the model prediction performance is shown in Table 6. In Table 6, some peptides exhibited completely different activities, despite their extreme sequence similarity, with some peptides even differing by only one amino acid sequence. The average accuracy of our proposed method on similar sequences is about 92.36%. HAN assigns different weights to the features of peptide sequences, which helps the model to focus on the most critical features step by step, so that the model still performs well for some sequences with extremely high similarity. However, some samples also have a poor predictive performance, with accuracy rates of only 86% for RYLG and 82% for RYPS, respectively. The amino acid sequence at both ends of the peptide has an important influence on the activity, so the activity prediction of similar sequences of CCK-secretory peptides is a difficult point in this study, and there is still room for improvement, to overcome this difficult point in the future.

## 4. Conclusions

In this study, a BiLSTM model (TL-AHB) based on transfer learning and a hierarchical attention network mechanism is proposed, for the first time, to predict CCK-secretory peptides. The SMILES enumeration method is used to enhance the SMILES, and the transfer learning strategy is combined to pre-train and fine-tune the model parameters. The information on the peptide chain is fully learned, and effective and ineffective peptides are recognized using the TL-AHB model. The experimental results demonstrate the excellent performance and robustness of the model. Of course, some difficulties still need to be overcome in the future. The model cannot measure the strength of the CCK-secretion-stimulating effect of peptides, and there are still broad research prospects for this topic. As an efficient and low-cost tool, the TL-AHB prediction model is expected to be used to predict and research CCK-secretory peptides. In addition, user-friendly and publicly accessible web servers are becoming a popular trend. This study aims to help biological researchers more easily identify CCK-secretory peptides, and we hope to provide a web server for the prediction method proposed in this paper in the future. A robust online web server can significantly enhance the influence of the TL-AHB model.

## Figures and Tables

**Table 1:** Details of the source dataset and target dataset used in this study.

Dataset	Name	Total	Positive	Negative
Source dataset	DILI	467	235	232
	Pgb-sub	1185	586	599
	SR-MMP	1840	892	948
Target dataset	CCK	99	54	45

**Table 2:** Performance comparison with different machine learning models developed based on AAC.

Model	Sen (%)	Spe (%)	Acc (%)	MCC (%)	AUC (%)
SVM	61.54	71.43	66.67	33.15	66.48
RF	76.92	64.29	70.37	41.44	70.60
MLP	69.23	57.14	62.96	19.56	59.62
KNN	61.54	50.00	55.56	11.60	55.77
GBDT	84.62	85.71	85.19	70.33	85.16
XGBoost	76.92	71.43	74.07	48.35	74.18

Sen: sensitivity, Spe: specificity, Acc: accuracy, MCC: Matthews correlation coefficient, AUC: the area under the receiver operating characteristic curve.

**Table 3:** Performance comparison with different machine learning models developed based on DPC.

Model	Sen (%)	Spe (%)	Acc (%)	MCC (%)	AUC (%)
SVM	61.54	18.18	57.14	30.93	59.09
RF	80.00	27.27	52.38	8.53	53.64
MLP	60.00	36.36	47.62	3.74	48.18
KNN	60.00	27.27	42.86	13.48	43.64
GBDT	90.00	9.09	47.62	1.55	49.55
XGBoost	40.00	72.73	57.14	13.48	56.36

Sen: sensitivity, Spe: specificity, Acc: accuracy, MCC: Matthews correlation coefficient, AUC: the area under the receiver operating characteristic curve.

**Table 4:** Performance of pre-training models on different source datasets.

Dataset	Model	Sen (%)	Spe (%)	Acc (%)	MCC (%)	AUC (%)
DILI	HL	77.33	84.80	80.46	60.47	85.84
	AHL	78.28	86.58	82.41	65.12	87.88
	HB	78.09	85.91	81.59	61.89	86.06
	AHB	85.66	81.16	83.34	67.19	90.05
pgb-sub	HL	79.37	70.01	73.92	48.80	83.17
	AHL	79.99	81.20	80.65	60.02	88.16
	HB	75.50	77.43	75.76	52.09	85.42
	AHB	85.54	80.08	82.28	64.59	89.90
SR-MMP	HL	80.10	82.20	81.04	61.96	88.60
	AHL	78.06	88.98	83.27	66.93	90.86
	HB	79.38	83.49	81.70	63.18	89.47
	AHB	79.77	87.97	83.81	67.66	91.13

A: using data augmentation; H: hierarchical attention network (HAN). L: LSTM. B: BiLSTM. Sen: sensitivity, Spe: specificity, Acc: accuracy, MCC: Matthews correlation coefficient, AUC: the area under the receiver operating characteristic curve.

**Table 5:** Performance of different transfer learning models on the target dataset.

Model	Sen (%)	Spe (%)	Acc (%)	MCC (%)	AUC (%)
TL-HL	74.36	82.93	80.17	55.94	83.58
TL-AHL	70.77	95.73	87.69	71.12	91.88
TL-HB	66.67	88.16	82.14	59.58	87.87
TL-AHB	90.71	98.92	95.99	91.27	98.07

TL: transfer learning. A: using data augmentation; H: hierarchical attention network (HAN). L: LSTM. B: BiLSTM. Sen: sensitivity, Spe: specificity, Acc: accuracy, MCC: Matthews correlation coefficient, AUC: the area under the receiver operating characteristic curve.

**Table 6:** Prediction performance of similar sequences using the model.

CCK-secretory Peptide Sequence	Class Label	Accuracy (%)
RYLGY [36]	1	96
SRYPS [36]	1	90
IRGCRL [36]	1	94
RYLG [36]	0	86
RYPS [36]	0	82
RGCRL [36]	0	94
LEL [37]	0	96
LLP [16]	0	96
LL [38]	0	96
IPI [38]	0	92
GPI [39]	0	94

Class label 1: effective peptides, Class label 0: ineffective peptides.

## Data Availability

Data are available from the corresponding author.
